# *In vitro* Anti-Thrombotic Activity of Extracts from Blacklip Abalone (*Haliotis rubra*) Processing Waste

**DOI:** 10.3390/md15010008

**Published:** 2016-12-31

**Authors:** Hafiz Ansar Rasul Suleria, Barney M. Hines, Rama Addepalli, Wei Chen, Paul Masci, Glenda Gobe, Simone A. Osborne

**Affiliations:** 1 School of Medicine, The University of Queensland, Translational Research Institute, Kent Street, Woolloongabba 4102, Australia; hafiz.suleria@uqconnect.edu.au (H.A.R.S.); p.masci@uq.edu.au (P.M.); 2 CSIRO Agriculture, 306 Carmody Road, St Lucia 4067, Australia; barney.hines@csiro.au (B.M.H.); rama.addepalli@csiro.au (R.A.); wei.chen@csiro.au (W.C.)

**Keywords:** blacklip abalone, processing waste, bioactive molecules, anti-thrombotic activity

## Abstract

Waste generated from the processing of marine organisms for food represents an underutilized resource that has the potential to provide bioactive molecules with pharmaceutical applications. Some of these molecules have known anti-thrombotic and anti-coagulant activities and are being investigated as alternatives to common anti-thrombotic drugs, like heparin and warfarin that have serious side effects. In the current study, extracts prepared from blacklip abalone (*Haliotis rubra*) processing waste, using food grade enzymes papain and bromelain, were found to contain sulphated polysaccharide with anti-thrombotic activity. Extracts were found to be enriched with sulphated polysaccharides and assessed for anti-thrombotic activity *in vitro* through heparin cofactor-II (HCII)-mediated inhibition of thrombin. More than 60% thrombin inhibition was observed in response to 100 μg/mL sulphated polysaccharides. Anti-thrombotic potential was further assessed as anti-coagulant activity in plasma and blood, using prothrombin time (PT), activated partial thromboplastin time (aPTT), and thromboelastography (TEG). All abalone extracts had significant activity compared with saline control. Anion exchange chromatography was used to separate extracts into fractions with enhanced anti-thrombotic activity, improving HCII-mediated thrombin inhibition, PT and aPTT almost 2-fold. Overall this study identifies an alternative source of anti-thrombotic molecules that can be easily processed offering alternatives to current anti-thrombotic agents like heparin.

## 1. Introduction

Marine organisms are increasingly being investigated as sources of bioactive molecules with therapeutic applications as nutraceuticals and pharmaceuticals [1]. Accordingly, processing waste from these organisms is an important source of bioactive molecules [2]. Abalone, a marine gastropod, contains a variety of bioactive molecules with reported anti-oxidant, anti-thrombotic, anti-inflammatory, anti-microbial and anti-cancer activities [3]. For thousands of years, different cultures have used abalone as a traditional functional food, believing that its consumption provides health benefits [4]. Recent research has revealed that abalone is composed of many bioactive molecules like sulphated polysaccharides, proteins and fatty acids that provide health benefits beyond basic nutrition [3]. In recent years abalone has been investigated as source of sulphated polysaccharides with anti-thrombotic activity with the potential to reduce thrombosis [5].

Thrombosis involves local clotting of blood in the vessel system that often leads to severe health related disorders like heart attack and stroke. The risk factors for thrombosis are abnormally high blood lipids, high blood glucose, elevated plasma fibrinogen, hypertension and cancer insurgence [6]. In the last several decades, prevention and treatment of thrombosis has been achieved with drugs, including heparin and warfarin. Heparin, a highly sulphated glycosaminoglycan (GAG) present in many mammalian tissues, is used commercially as an anti-coagulant or anti-thrombotic drug [7]. Heparin is administered intravenously, with frequent laboratory monitoring needed to prevent unwanted and sometimes life-threatening bleeding [8]. Heparin-induced thrombocytopenia (a low platelet count) is another serious complication following heparin therapy, particularly in some cardiac patients [9]. Heparin also has other disadvantages as it is extracted and purified from bovine and porcine internal organs making its production difficult and prone to contamination by other GAGs present in these sources [10].

These disadvantages have necessitated a field of research aimed at discovering novel anti-thrombotic and anti-coagulant agents with fewer side effects than heparin. Heparin-like molecules are present in lower invertebrates [11], lobster [12], ascidians and tunicates [13]. Many other marine species including molluscs, that are rich in sulphated polysaccharides, contain GAG-like molecules that have comparable biological activity to heparin [14]. These uniquely sulphated polysaccharides have complex structures composed of galactose, fucose, glucuronic acid and galactosamine. Sulphated polysaccharides isolated from molluscs were found to contain anti-thrombin and anti-coagulant bioactive molecules with unique 3-*O*-sulphated glycosamine residues [11]. In general, these sulphated polysaccharides significantly vary between species with respect to their composition. Furthermore, bioactivity has also been found to differ depending upon the degree of sulphation, molecular weight, types of saccharides present and glycosidic branching [15].

Many studies have demonstrated abalone viscera, gonads and pleopods are sources of potent anti-thrombotic and anti-coagulant polysaccharides, however limited research has been conducted *in vitro* using plasma and blood to investigate the anti-thrombotic and anti-coagulant mechanisms [8,11,14,15]. Several well-established analyses are used to indicate anti-thrombotic activity including prothrombin time (PT), activated partial thrombin time (aPTT) and thromboelastography (TEG). These assays help to indicate if molecules act in both the intrinsic and extrinsic pathways of the blood coagulation cascade [16] and what the impact on platelets might be. The aim of the current research was to extract, purify and characterise GAG-like sulphated polysaccharides from offal samples from processed wild caught *H. rubra* and assess anti-thrombotic and anti-coagulant activity in the extracts using *in vitro* plasma and blood assays.

## 2. Results and Discussion

### 2.1. Protein and Sulphated Polysaccharide Content of Extracts from Blacklip Abalone Processing Waste

Table 1 shows the protein and sulphated polysaccharides that were estimated in all crude extracts and expressed as mg sulphated polysaccharides or protein per gram of starting abalone processing waste material (wet weight). Sulphated polysaccharides were similar across all samples, however protein content was found to be significantly higher in canned abalone extracts compared to the liquid abalone extracts, particularly following enzymatic treatment with papain, and with papain and bromelain combined.

There were no sulphated polysaccharides detected in any enzyme (empty digest) control. However as expected, protein was detected in all enzyme controls (data not shown). Simultaneous digestion with papain and bromelain produced higher contents of sulphated polysaccharides in both canned and liquid samples compared to single enzyme digestions, however these differences were not statistically significant. Initial screening of anti-thrombin activity mediated by heparin cofactor-II (HCII) depicted that most of the thrombin inhibition arose directly from the abalone samples with enzyme control contributing less than 10% of the total thrombin inhibition.

The presence of sulphated polysaccharides in abalone processing waste has been observed previously in other abalone species [17]. The extraction and purification of these sulphated polysaccharides can be achieved by different processes, including enzyme digestion. Different enzymes have different hydrolysing activities that vary in efficiency depending upon sample type, time of incubation, pH and buffer [18]. Kechaou et al. [19] digested cuttlefish and sardine viscera with several commercial proteases, including papain. In this study the degree of hydrolysis for cuttlefish was higher than that obtained for sardine. The authors speculated that the differences may be due to a difference in protein composition of the tissues and nature of the samples influencing the digestion profile. In short, enzyme digestion varies depending upon the nature of the sample and hydrolysing conditions, however it appears as though papain is commonly used in the digestion of various marine samples.

In the study presented here, combined enzymatic treatment with 0.5% *w/v* papain and 0.5% *w/v* bromelain liberated the highest levels of sulphated polysaccharides and protein from canned abalone processing waste compared to separate treatments with 1% *w/v* papain or 1% *w/v* bromelain. These results suggest that the different protease activities of bromelain and papain are required to produce optimal release of sulphated polysaccharides and protein from abalone processing waste. Regardless of enzyme treatment, all extracts were investigated for anti-thrombotic and anti-coagulant activity.

### 2.2. Separation of Abalone Extracts Using Anion Exchange Chromatography-Fast Performance Liquid Chromatography (AEC-FPLC)

**Figure 1:**
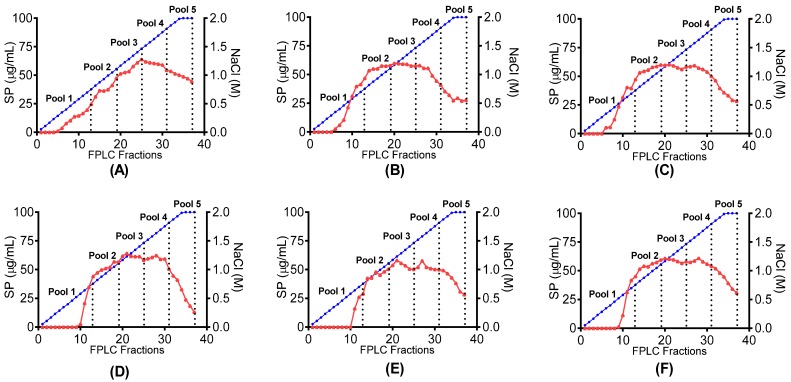
AEC-FPLC chromatograms showing separation of abalone extracts through interaction with DMMB. Concentration of sulphated polysaccharide (SP) 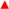 is shown in relation to linear NaCl gradient 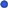 in canned abalone processing waste digested with (**A**) papain; (**B**) bromelain; (**C**) papain + bromelain, and liquid abalone processing waste digested with (**D**) papain; (**E**) bromelain and (**F**) papain + bromelain.

Based on previous studies it was hypothesised that sulphated polysaccharides present in the abalone processing waste may confer both anti-thrombotic and anti-coagulant activity. To produce fractions from the abalone extracts with enhanced sulphated polysaccharide content and anti-thrombotic and anti-coagulant activity, all extracts were subjected to an anion exchange chromatography-fast performance liquid chromatography (AEC-FPLC) system. As shown in Figure 1, separation into sulphated polysaccharide-containing fractions was monitored through their interactions with 1,9-dimethylene blue (DMMB) dye. Similar elution profiles were observed for almost all extracts where sulphated polysaccharide concentration was mostly lower in first 10 eluted fractions (0.4 M NaCl) and generally increased until fraction 30 (1.6 M NaCl). For analysis, seven AEC pools were prepared. Five AEC pools were prepared from the NaCl gradient (AEC pools 1–5), whilst the initial unbound material and a final column wash were also collected and analyzed for bioactivity along linear gradient pools. All three enzyme controls showed similar elution profiles. Most of the peptides eluted during sample application while few peptides were detected in AEC Pool 1. However, there was no protein or sulphated polysaccharides detected after AEC Pool 1 in all three enzyme controls indicating no binding with column. Finally, to decrease NaCl concentration in all pools, 3 kDa spin columns and deionised water washes were performed until the NaCl concentration was below 60 mM.

Protein and sulphated polysaccharides concentrations (via Blyscan™ Sulphated GAG assay) were estimated in all the AEC pools and are shown in Table 2. The highest sulphated polysaccharide concentrations were mostly measured in the gradient AEC pools 3 and 4 (0.8–1.0 M NaCl) whereas the highest protein concentrations were measured in the unbound material because of almost complete elution of enzymes. Some of the enzyme peptides were also detected in early AEC pool 1 during ion exchange chromatography of empty control/enzyme controls.

In similar separation studies, different types of sulphated polysaccharides have been obtained from visceral portions and gonads of abalone [20]. Wang et al. [21] isolated and characterized three sulphated polysaccharides, AAP, AVAP I and AVAP II, from the pleopods and viscera of the abalone *H. discus hannai* Ino. The crude polysaccharide extract was initially separated by AEC on a diethylaminoethyl-cellulose (DEAE-cellulose) column with the main polysaccharide fraction from the pleopods eluted with 0.42–0.60 M NaCl, whilst two fractions from the viscera eluted with 0.28–0.40 M NaCl and 0.44–0.56 M NaCl.

Zhu et al. [22] demonstrated that sulphated polysaccharides isolated from pleopods of abalone consist of 1-1,4-, 1,6-, or 1,4,6-linked glucose, and in accretion 1-, 1,3-, 1,6-, and 1,4,6-linked galactose. Prior to this, She [23] also proposed that sulphated polysaccharides from abalone pleopods are comprised of galactose, glucose, fructose and xylose. The acidic polysaccharide content has not been fully determined, however several GAG-like structures have been defined in abalone. The chemical structure of sulphated polysaccharides, isolated by Li et al. [17], contains a galactosamine and glucuronic acid backbone linked to sulphated-fucose and galactose, considered to be similar to the fucosylated chondroitin sulphate present in the sea cucumber [24]. In abalone, even though these sulphated polysaccharides are linked with galactose to the fucose branch, it is still considered a chondroitin-like polysaccharide [17].

Based on the assumption that the sulphated polysaccharides present in abalone processing waste are also GAG-like molecules, the anti-thrombotic and anti-coagulant activities were investigated using several *in vitro* assays.

### 2.3. Anti-Thrombotic Activity Measured through HCII-Mediated Thrombin Inhibition

To determine the anti-thrombotic activity of the different abalone processing waste extracts and AEC pooled fractions, HCII-mediated thrombin inhibition was measured using the chromogenic substrate Chromozym TH. All samples were initially screened without dilution to determine which extracts and AEC pooled fractions contained anti-thrombotic molecules (data not shown). No activity was observed in the enzyme controls, AEC Pool 1 and in the final column washes (data not shown). The extracts and AEC pooled fractions that produced anti-thrombotic activity were then examined further in order to compare the *in vitro* HCII-mediated thrombin inhibition between the different samples. *In vitro* HCII-mediated anti-thrombin activity, expressed as percentage inhibition of thrombin, is presented in Table 3 and shows that AEC pools 4 or 5 displayed the highest activity. AEC Pool 4 of all abalone extracts showed significantly higher inhibition of thrombin (*p* < 0.05) relative to other pools at a sulphated polysaccharide concentration of 100 μg/mL.

It was also observed that most of the time AEC pool 4 displayed highest activity at 10 and 1 μg/mL sulphated polysaccharide concentration. Moreover, specific enzyme digestion also showed a marked effect on percentage inhibition; papain treatment alone or in combination appeared to be the most efficient enzyme with respect to release of sulphated polysaccharides, whilst bromelain digestion inconsistently inhibited thrombin and generally produced the lowest percentage inhibition. The activity of the AEC pool 4 of the liquid abalone sample digested with papain and bromelain showed thrombin inhibition only 2–3 times less compared to the heparin standard (on a similar sulphated polysaccharides basis).

Other studies involving sulphated polysaccharides from marine sources support the anti-thrombotic effects observed in this study [25]. These studies also proposed different mechanisms of action involving factors Xa and thrombin (IIa) mediated by HCII and anti-thrombin III (ATIII) [26]. The specific pattern of sulphation and the position of glycosidic linkages varied among different species and may contribute to difference in activity [27]. For example, sea cucumber polysaccharides displayed weaker anti-factor Xa and anti-thrombin activities mediated by ATIII compare to both heparin and low molecular weight heparin, suggesting that their anti-coagulant mechanisms are different from those of heparin-like drugs. Thus, the structural interaction of these polysaccharides with coagulation cofactors (HCII and ATIII) and their target proteases may be influenced by the conformation and length of repetitive sulphated units [28].

Based on previous reports and on the findings presented here, extracts as well as the AEC pooled fractions 3–5 were selected for further assessment using PT, aPTT and TEG assays to confirm the anti-thrombotic and anti-coagulant effect of these samples in blood and plasma and to help elucidate the role of these molecules in the coagulation cascade.

### 2.4. Anti-Thrombotic and Anti-Coagulant Activity in Blood and Plasma

#### 2.4.1. Prothrombin and Activated Partial Thromboplastin Time

**Figure 2:**
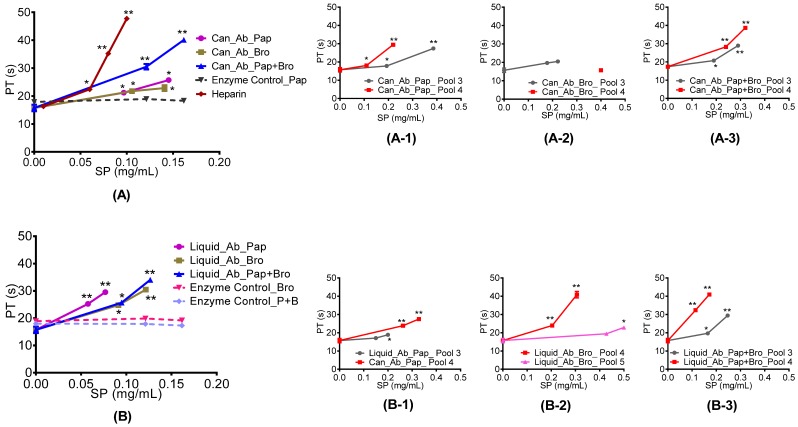
Prothrombin time (PT) of abalone processing waste extracts and AEC pooled fractions. Graphs (**A**, **A-3**): canned abalone processing waste digested with papain, bromelain and a combination of papain and bromelain and their respective AEC pools 3 and 4; (**B**, **B-3**): liquid abalone processing waste digested with papain, bromelain and a combination of papain and bromelain and their respective AEC pools 3 and 4. * = Statistical significance determined using a one way ANOVA with Dunnett’s Multiple Comparison Test compared to saline control with * *p* < 0.05 and ** *p* < 0.01.

**Figure 3:**
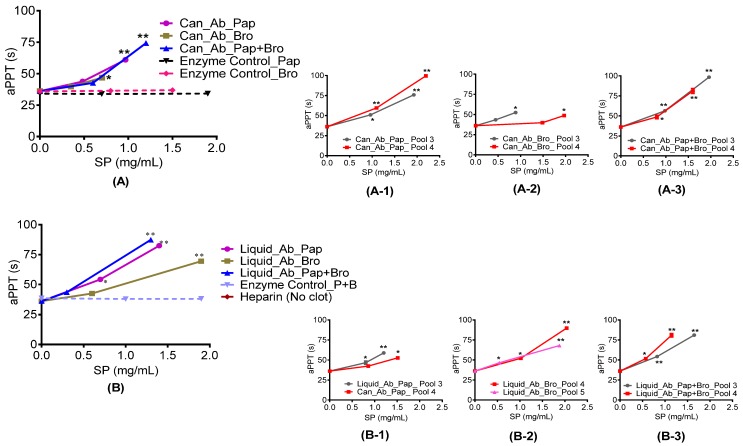
Activated partial thrombin time (aPTT) of abalone processing waste extracts and AEC pooled fractions. (**A**, **A-3**) canned abalone digested with papain, bromelain and a combination of papain and bromelain and their respective AEC pool 3 and 4 while (**B**, **B-3**)**,** liquid abalone samples digested with papain, bromelain and a combination of papain and bromelain and their respective AEC pool 3 and 4. * Statistical significance determined using a one way ANOVA with Dunnett’s Multiple Comparison Test compared to saline control with * *p* < 0.05 and ** *p* < 0.01. Heparin standard did not form a clot even at very low concentration. It showed that the heparin standard is many times stronger in aPTT activity compare to abalone extract and anion exchanged pools.

The *in vitro* clotting assays PT and aPTT showed positive responses to all abalone extracts and pools 3 and 4. Figure 2 demonstrates that PT increased with sulphated polysaccharide concentration, and that pools 3 and 4 generally prolonged PT time more effectively than the original extracts. In particular, pool 4 from both the canned and liquid abalone samples significantly increased PT compared with other pools. Comparing to heparin standard, both extract and pools showed an increase in PT activity. PT and aPTT were not observed in the enzyme controls. The aPTT also showed an increase with sulphated polysaccharide concentration. In Figure 3, both canned and liquid extracts, digested with different enzymes, prolonged aPTT significantly compared to the saline control.

Furthermore, all three enzyme controls were also subjected to aPTT analysis and they were unable to prolong aPTT. Moreover, heparin standard was also subjected to aPTT analysis. Even at a very low concentration of 0.02 mg/mL, no clot formed, demonstrating that heparin standard has a very strong aPTT activity and it can prolong aPTT many fold higher compared with our extract. However, from the results it appeared that AEC pools 3 and 4 prolonged aPTT more compared with the original extract. Furthermore, AEC pool 4 from both the canned and liquid abalone processing waste appeared to prolong aPPT more when compared to the remaining AEC pools.

The results presented here are supported by several other studies that report the pharmaceutical importance of abalone. Previously, Li et al. [17] isolated a GAG-like polysaccharide from abalone, and conducted *in vitro* investigations on its anti-coagulant activity. Usually *in vitro* anti-coagulant assays thrombin time (TT), PT and aPTT and help to indicate anti-coagulant activity with respect to the intrinsic and extrinsic pathways in the blood coagulation cascade. PT reflects the extrinsic pathway of the coagulation cascade whilst aPTT reflects changes in the intrinsic pathway of the blood coagulation cascade [16]. Li et al. [17] found that the GAG-like polysaccharide could prolong aPTT as well as TT.

In another study by Li et al. [29], different types of extracts were prepared from abalone viscera and it was found that water extracts were associated with higher PT, aPTT and TT when compared to extracts prepared using different solvents. The heparin control displayed higher specific activity in processing waste from abalone offal. Other research also demonstrated that GAG-like molecules isolated from different sea cucumbers did not show a prominent effect on PT but efficiently improved the aPTT and TT. It also indicated that the type of sulphated polysaccharides present in sea cucumber may affect the intrinsic but not the extrinsic and common coagulation process [24] compared with abalone. This suggests abalone sulphated polysaccharides are involved in both intrinsic and extrinsic pathways. In order to confirm this mode of action and further demonstrate a role for abalone sulphated polysaccharides in both intrinsic and extrinsic pathway, an *in vitro* blood assay was performed using TEG.

#### 2.4.2. Thromboelastography (TEG)

TEG is a global assessment of haemostatic function investigating the interaction of platelets with the coagulation cascade from the time of initial fibrin formation through to platelet aggregation, clot strengthening, fibrin cross linkage, and to eventual clot lysis. To assess anti-coagulant activity in this study, TEG parameters including R-time, α-angle and MA value were measured.

All extracts and AEC pooled fractions were freeze dried, resuspended in deionised water and added to whole blood in the TEG assay. Table 4 demonstrates that both liquid and canned abalone samples have anti-coagulant activity. In particular, R time is prolonged significantly when sulphated polysaccharide concentrations are increases compared to the saline control. Higher anti-coagulant activity was associated with stronger effects on α-angle and MA values that decreased significantly compared to saline control. These results suggest that molecules present in abalone extracts have an effect on clot strength and platelet function indicating that the kinetics of fibrin polymerization and networking are also affected.

The AEC pool fractions prolonged R time more than the original extracts. Generally, AEC pool 3 and 4 appeared to increase R time more than other AEC pools (data not shown). In these samples, R time increased with an increasing sulphated polysaccharide concentration. However it was also observed that both fractions from AEC pool 3 and 4 affected MA values and α-angles more than the extracts, suggesting a greater effect on clot strength and platelet function because α-angles indicate fibrin build up and cross-linking while MA values reflect clot formation, firmness and platelet function.

The relationship between structure and anti-coagulant activity has been previously investigated in detail for heparin and fucosylated chondroitin sulphate [24]. Both molecules inhibit the intrinsic and/or common pathways of coagulation and thrombin activity or conversion of fibrinogen to fibrin, as observed in the study presented here. This is in agreement with a TEG study reported by Fischer et al. [30] who demonstrated that a variety of glucosamine-based biopolymers including the marine-derived poly-*N*-acetyl glucosamine could decrease the R time and increase maximal clot strength in plasma. The haemostatic properties were highly dependent on the chemical nature and tertiary/quaternary structure of these biomaterials.

## 3. Materials and Methods

### 3.1. Chemicals

All chemicals were reagent grade and unless otherwise indicated were from Sigma-Aldrich (St Louis, MO, USA). Food grade enzymes papain 30,000 (30,000 PU/gram, Papain Units) and bromelain concentrate (2400 GDU/gram, Gelatin Digestion Units) were from Enzyme Solutions Pty Ltd. (Croydon South, Australia). The Blyscan™ Glycosaminoglycan Assay was from Biocolor (Carrickfergus, County Antrim, UK). Other suppliers are listed here: Pierce BCA Protein Assay kit (Quantum Scientific, Murarrie, Australia); Q Sepharose™ Big Beads (GE Healthcare Life Science, Chicago, IL, USA); Thromborel S^®^ (Dade Behring Inc. Newark, NJ, USA); Triniclot (Haemostasis, Wicklow, Ireland); Chromozym TH (Roche Diagnostics, Basel, Switzerland); and human alpha-thrombin and human Heparin Cofactor II (HCII) (US Biologicals, Salem, MA, USA).

### 3.2. Preparation of Extracts

Processed wild caught *H. rubra* byproducts (comprised of viscera and gonads) were provided by Lonimar Australia Pty Ltd (formerly of Melbourne, Australia). Samples were received either as a paste (canned) or liquid (hot-filled bag). All samples were stored at −20 °C.

Extracts were prepared using the food grade proteases papain and bromelain either separately or combined as a 1:1 ratio. Each digest contained 20 g abalone processing waste and 1% *w/v* papain or bromelain, or a mixture of 0.5% *w/v* papain and 0.5% *w/v* bromelain, in water (final volume 100 mL). For the purpose of determining the contribution of the two enzymes to the subsequent assay measurements, enzyme-only control digests were prepared using the same concentrations and conditions but with no added abalone extract. Digests were incubated overnight (14–16 h) at 50 °C, inactivated by heating at 95 °C for 10 min, cooled on ice and centrifuged (Beckman Coulter, Avanti^®^ J-26XP1, Brea, CA, USA) at 5940× *g* for 10 min to remove undigested material (pellet). Approximately 0.6 g of undigested material was usually discarded. Supernatants were clarified using 2, 1 μm (Whatman™, GE Healthcare, Life Science, Chicago, IL, USA) and 0.45 μm filtration (mixed cellulose ester, Merck Millipore, Bayswater VIC, Australia) and extracts were stored at −20 °C. Due to likely interference with bioassays, salt ions were removed from all abalone extracts and pooled fractions using 3 kDa molecular weight cut off (MWCO) spin columns (Centrifuge Filter Unit, Merck Millipore, Billerica, MA, USA). 10 mL samples were added into the spin column and centrifuged at 3270× *g* for 30 min. This process was repeated using deionised water until salt was at or below 60 mM. Conductivity was measured using a Metler Toledo-AG conductivity meter (VWR International Pty, Ltd., Dietikon, Switzerland). Salt concentration was extrapolated using a NaCl standard curve.

### 3.3. Estimation of Sulphated Glycosaminoglycan Content

#### 3.3.1. Dimethyl-Methylene Blue (DMMB) Assay

Sulphated polysaccharide concentration was initially estimated in samples and extracts through interaction with DMMB dye. 200 μL DMMB dye solution was added to 25 μL samples, blanks or standards (chondroitin sulphate from bovine trachea, Sigma-Aldrich, Castle Hill, NSW, Australia) in triplicate, in a 96 well plate (Nunclon Delta Surface, Thermo Fisher Scientific, Waltham, MA, USA). The plate was mixed for 1 min using a plate mixer (IKA^®^ MS digital 96 Well Plate Mixer, Staufen im Breisgau, Germany) and absorbance was measured at 525 nm using a Spectra-Max M3 System spectrophotometer (Molecular Devices, Sunnyvale, CA, USA). Sulphated polysaccharides were calculated from the standard curve using SoftMax-Pro 6.1 software (Molecular Devices, Sunnyvale, CA, USA).

#### 3.3.2. Blyscan Sulphated Glycosaminoglycan (GAG) Assay

Sulphated polysaccharide concentration was measured in all samples and pooled fractions using the Blyscan™ Sulphated Glycosaminoglycan assay according to manufacturer’s instructions (Biocolor Ltd., Carrickfergus, County Antrim, UK), with modifications. Blyscan Dye Reagent (250 μL) was added to 25 μL sample, blank or standard (chondroitin sulphate from bovine trachea, Sigma-Aldrich, Castle Hill, NSW, Australia) in triplicate, in a 96 well V-bottom plate (Stor Plate-96 V-bottom, Perkin Elmer, Waltham, MA, USA). The plate was placed on an orbital shaker for 30 min (500 rpm) followed by centrifugation at 3270× *g* (Beckman Coulter, Allegra™ X-12R, Lane Cove, NSW, Australia) for 10 min. Supernatant was removed without disturbing the pellet using a vacuum before 250 μL Blyscan Dye Dissociation Reagent was added to each well. The plate was again placed on the orbital shaker for 30 min (500 rpm) or until complete dissociation of the pellet. 200 μL of the resuspended solution was transferred from each well into a 96 Well Plate to enable absorption to be measured at 656 nm in a Spectra-Max M3 System spectrophotometer. Sulphated polysaccharides were calculated from the standard curve using SoftMax-Pro 6.1 software.

### 3.4. Estimation of Protein Content

Protein content was estimated in all samples and extracts using the Pierce BCA Protein Assay Kit (Thermo Fisher Scientific, Waltham, MA, USA) with bovine serum albumin (BSA) as a protein standard, according to manufacturer’s instructions. Absorbance was measured at 562 nm using a Spectra-Max M3 System spectrophotometer. Protein concentration was calculated from the standard curve using SoftMax-Pro 6.1 software.

### 3.5. Separation of Extracts Using Anion Exchange Chromatography-Fast Performance Liquid Chromatography (AEC-FPLC)

An empty 200 mm × 16 mm column (GE-XK 16/20) packed with 13.5 mL Q Sepharose™ Big Beads and connected to fast protein liquid chromatography (FPLC) system (ÄKTA Lab-Scale Systems, GE Healthcare Life Science, Chicago, IL, USA) was used to fractionate the abalone extracts on the basis of their anionic interactions. The column was equilibrated with deionised water (Buffer A) before approximately 28 mg sample (based on sulphated polysaccharides as measured by DMMB assay) was loaded onto the column. Flow rate was set at 5 mL/min with a column pressure of 1 MPa. Fractions (2 mL each) were collected using a 0–2 M NaCl linear gradient over 20 min. AEC fractions were collected and pooled (7 pools for each sample) on the basis of their interactions with DMMB dye. For further analysis, all AEC pooled samples were desalted using 3 kDa MWCO spin columns and washed using deionised water.

### 3.6. Assessment of Anti-Thrombotic and Anti-Coagulant Activity

#### 3.6.1. Heparin Cofactor II (HCII) Mediated Thrombin Inhibition Assay

*In vitro* HCII-mediated thrombin inhibition was measured in the extracts and AEC pooled fractions using a kinetic assay as previously described by Dupouy et al. [31] with modifications by Hines et al. [32]. Briefly, 12.9 μM HCII in 0.02 M Tris-HCl pH 7.4/0.15 M NaCl/1 mg/mL polyethylene glycol (PEG) and 1 μL serially diluted extract, sample or standard (heparin from porcine intestinal mucosa, Sigma-Aldrich) was added to a 384 well plate (SpectraPlate-384 TC, clear, tissue culture with lid, Perkin Elmer, Waltham, MA, USA) by an epMotion^®^ 5075L/epMotion^®^ 5075TMX automated pipetting system (Eppendorf, Hamburg, Germany), mixed and incubated at room temperature for 22 min. Thrombin was then added (0.45 μM) prior to the final addition of 83 μM Chromozym TH. The assay was incubated at 37 °C for 40 min with absorbance measured at 405 nm every 2 min in a Spectra-Max M3 System spectrophotometer. HCII-mediated thrombin inhibition was measured at 10 min in triplicate and expressed as the mean percentage inhibition of thrombin activity ± standard error.

#### 3.6.2. Prothrombin Time (PT) Assay

To measure PT, 100 μL citrated plasma was added to a glass clotting tube and incubated at 37 °C on the heating block of a Hyland-Clotek clotting machine for 5 min. 50 μL saline (negative control), heparin/Clexane (positive control) or diluted abalone extracts or AEC pooled fractions were added to the tube. The volume was adjusted to 150 μL with plasma before the final addition of 100 μL Thromborel S^®^ (Dade Behring Inc. Newark, NJ, USA) to initiate clotting. Time in seconds until clot formation was measured in triplicate and expressed as the mean ± standard error.

#### 3.6.3. Activated Partial Thromboplastin Time (aPTT) Assay

To measure aPTT, 100 μL citrated plasma, 100 μL Triniclot (Haemostasis, Wicklow, Ireland) and diluted abalone extracts and AEC pooled fractions were added to a clotting tube. The final volume was adjusted to 250 μL with saline. The clotting tube was incubated at 37 °C in a heating block (Hyland-Clotek clotting machine) for 5 min before 50 μL 50 mM calcium solution in saline were added to initiate clotting. Time in seconds until clot formation was measured in triplicate and expressed as the mean ± standard error.

#### 3.6.4. Thromboelastography (TEG)

**Figure 4:**
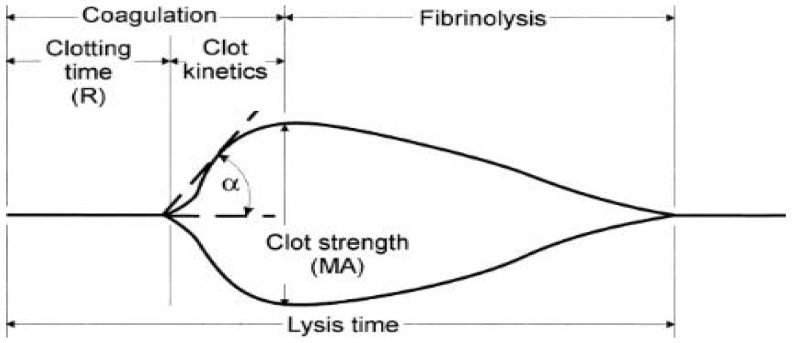
Representation of a typical TEG trace obtained during the clotting of citrated whole blood [33].

To measure clot dynamics in the presence of abalone extracts and AEC pooled fractions, TEG (Haemonetics, Braintree, MA, USA) analyses were undertaken. These analyses provide measurements of whole blood hemostasis that help to assess bleeding and thrombotic risks, as well as monitor anti-thrombotic therapies by investigating the shear elasticity of a clot as it forms or lyses through the following parameters: reaction time (R) which is the time from the start of a sample run until the first detectable clot formation (this is the point at which most traditional plasma clotting assays reach their end point); α-Angle (α) which is the measurement of the rapidity of fibrin build-up and cross-linking (or clot strengthening); and maximal amplitude (MA) which is the maximal stiffness or strength (maximal shear modulus) of the developed clot. A typical TEG tracing, generated by the TEG companion software is shown in Figure 4 and depicts R, α and MA. The parameters are as labelled and described by the TEG^®^ (Haemostasis Analyser, Braintree, MA, USA) 5000 Series Manual.

In this method, 280 μL citrated whole blood and 20 μL 0.2 M CaCl_2_, along with 20 μL of the negative (saline) or positive (heparin) controls or the abalone test compounds, were added into a disposable TEG cup (Haemonetics, Braintree, MA, USA).

### 3.7. Statistical Analyses

All statistical analyses were conducted using a one-way ANOVA with post hoc comparisons using Tukey’s multiple comparison test. These calculations were carried out using GraphPad Prism 5 Software for Windows (GraphPad 5 Software, San Diego, CA, USA, www.graphpad.com).

## 4. Conclusions

Extracts were prepared from blacklip abalone processing waste using food grade proteases and separated by AEC to produce fractions enriched with sulphated polysaccharides. These sulphated polysaccharides appeared to display properties similar to GAG-like molecules previously characterised in other abalone species by inhibiting thrombin activity through HCII and displaying significant anti-coagulant activity in plasma and blood. Further studies are needed to improve our understanding of the anti-coagulant mechanism *in vitro* and the critical structures required for the mechanism/s behind the anti-coagulant activity.

## Figures and Tables

**Table 1:** Protein and sulphated polysaccharide content of extracts from abalone processing waste expressed in mg per gram (on a wet weight basis).

Abalone Waste	Treatments	Protein (mg/g)	Sulphated Polysaccharides (mg/g)	Anti-Thrombin HCII (% Inhibition)
Canned	Papain	29.95 ± 0.51 ^b^	1.36 ± 0.09 ^a^	92.1 ± 1.31 ^b^
Canned	Bromelain	25.06 ± 1.79 ^c^	1.39 ± 0.91 ^a^	89.9 ± 2.09 ^c^
Canned	Papain + Bromelain	36.10 ± 0.72 ^a^	1.46 ± 0.38 ^a^	96.8 ± 1.12 ^a^
Liquid	Papain	18.82 ± 0.10 ^e^	1.27 ± 0.82 ^a^	97.1 ± 0.08 ^a^
Liquid	Bromelain	23.38 ± 2.09 ^d^	1.03 ± 0.13 ^a^	95.4 ± 2.13 ^a^
Liquid	Papain + Bromelain	18.90 ± 0.80 ^e^	1.41 ± 0.68 ^a^	91.1 ± 0.79 ^b^

Alphabetic letters shows the difference between different samples and treatments. HCII, heparin cofactor-II.

**Table 2:** Protein and sulphated polysaccharide concentration of pooled fractions from AEC-FPLC abalone extracts.

Sample Descriptions	Protein (mg/mL)	Sulphated Polysaccharides (mg/mL)
Can_Ab_Pap_Unbound material	3.40 ± 1.1	1.12 ± 0.9
Can_Ab_Pap_AEC Pool 1	0.76 ± 0.7	0.19 ± 0.4
Can_Ab_Pap_AEC Pool 2	0.59 ± 0.4	1.04 ± 0.2
Can_Ab_Pap_AEC Pool 3	1.87 ± 0.2	1.82 ± 1.2
Can_Ab_Pap_AEC Pool 4	0.37 ± 0.1	1.49 ± 0.2
Can_Ab_Pap_AEC Pool 5	0.23 ± 0.9	1.15 ± 1.3
Can_Ab_Pap_Final column wash	0.36 ± 2.3	0.03 ± 2.1
Can_Ab_Bro_Unbound material	5.77 ± 1.4	0.56 ± 0.9
Can_Ab_Bro_AEC Pool 1	0.74 ± 0.4	0.30 ± 0.3
Can_Ab_Bro_AEC Pool 2	0.64 ± 0.1	1.02 ± 0.8
Can_Ab_Bro_AEC Pool 3	0.16 ± 0.2	1.11 ± 0.4
Can_Ab_Bro_AEC Pool 4	0.11 ± 0.1	1.21 ± 0.1
Can_Ab_Bro_AEC Pool 5	0.05 ± 1.2	0.22 ± 1.1
Can_Ab_Bro_Final column wash	0.06 ± 2.1	0.02 ± 2.1
Can_Ab_Pap+Bro_Unbound material	8.92 ± 1.2	0.65 ± 0.4
Can_Ab_Pap+Bro_AEC Pool 1	1.31 ± 0.3	0.42 ± 0.2
Can_Ab_Pap+Bro_AEC Pool 2	1.90 ± 0.1	2.01 ± 1.1
Can_Ab_Pap+Bro_AEC Pool 3	0.37 ± 0.7	2.45 ± 0.9
Can_Ab_Pap+Bro_AEC Pool 4	0.33 ± 0.1	2.00 ± 0.1
Can_Ab_Pap+Bro_AEC Pool 5	0.05 ± 0.3	0.38 ± 0.7
Can_Ab_Pap+Bro_Final column wash	0.67 ± 0.9	0.54 ± 1.2
Liquid_Ab_Pap_Unbound material	2.52 ± 1.1	0.03 ± 0.4
Liquid_Ab_Pap_AEC Pool 1	0.17 ± 0.2	0.04 ± 0.1
Liquid_Ab_Pap_AEC Pool 2	0.44 ± 0.8	0.70 ± 0.8
Liquid_Ab_Pap_AEC Pool 3	0.33 ± 0.3	1.25 ± 0.7
Liquid_Ab_Pap_AEC Pool 4	0.13 ± 0.1	1.64 ± 0.2
Liquid_Ab_Pap_AEC Pool 5	0.02 ± 1.2	0.16 ± 0.7
Liquid_Ab_Pap_Final column wash	0.39 ± 2.1	0.01 ± 1.1
Liquid_Ab_Bro_Unbound material	6.12 ± 1.1	0.40 ± 0.9
Liquid_Ab_Bro_AEC Pool 1	0.00 ± 2.1	0.01 ± 0.1
Liquid_Ab_Bro_AEC Pool 2	0.00 ± 0.5	0.02 ± 0.9
Liquid_Ab_Bro_AEC Pool 3	0.91 ± 0.1	1.04 ± 0.2
Liquid_Ab_Bro_AEC Pool 4	1.41 ± 0.5	3.10 ± 0.7
Liquid_Ab_Bro_AEC Pool 5	0.21 ± 1.2	0.61 ± 1.1
Liquid_Ab_Bro_Final column wash	0.11 ± 0.9	0.47 ± 1.9
Liquid_Ab_Pap+Bro_Unbound material	10.34 ± 0.9	0.95 ± 0.5
Liquid_Ab_Pap+Bro_AEC Pool 1	0.45 ± 0.7	0.06 ± 0.6
Liquid_Ab_Pap+Bro_AEC Pool 2	0.39 ± 0.9	2.07 ± 0.7
Liquid_Ab_Pap+Bro_AEC Pool 3	0.56 ± 0.3	2.06 ± 0.2
Liquid_Ab_Pap+Bro_AEC Pool 4	0.27 ± 0.2	1.43 ± 0.1
Liquid_Ab_Pap+Bro_AEC Pool 5	0.11 ± 0.1	0.53 ± 0.9
Liquid_Ab_Pap+Bro_Final column wash	0.10 ± 1.1	0.05 ± 0.1

“Ab” stands for abalone, “Pap” stands for papain enzyme, “Bro” stands for bromelain enzyme while “Pap+Bro” reflects the combination of both papain and bromelain enzymes.

**Table 3:** Heparin cofactor II-mediated thrombin inhibition by abalone samples expressed as percentage inhibition of thrombin activity.

Percentage Inhibition of Thrombin Mediated by HCII at 10 min				
Sample Description	Sulphated Polysaccharide Concentration (μg/mL)			
	100	50	10	1
Can_Ab_Pap	93.1 ± 0.8 ^d^	26 ± 7.5 ^j^	11.4 ± 10.2 ^l^	0
Can_Ab_Pap_Unbound material	56 ± 0.4 ^k^	26.9 ± 0.8 ^j^	0	0
Can_Ab_Pap_AEC Pool 2	8.5 ± 1.9 ^s^	8.7 ± 3.4 ^q^	0	0
Can_Ab_Pap_AEC Pool 3	87.6 ± 3.2 ^e^	81.8 ± 0.8 ^f^	13.9 ± 6.6 ^k^	0
Can_Ab_Pap_AEC Pool 4	96.1 ± 0.4b ^c^	92.7 ± 1.7 ^c^	43 ± 3.9 ^f^	9.4 ± 6.6 ^d^
Can_Ab_Pap_AEC Pool 5	97.5 ± 0.2 ^ab^	94.6 ± 1.8 ^b^	78.2 ± 2.7 ^b^	20.7 ± 4.2 ^b^
Can_Ab_Bro	72.3 ± 1.2 ^g^	13.2 ± 2.9 °	2.4 ± 1.1 ^p^	0
Can_Ab_Bro_Unbound material	14.2 ± 5.1 ^r^	0	0	0
Can_Ab_Bro_AEC Pool 2	56.6 ± 1.9 ^jk^	38.5 ± 6.1 ^h^	0	0
Can_Ab_Br_AEC Pool 3	26.4 ± 3.7 ^q^	21.5 ± 3.6 ^m^	3.6 ± 4.5 °	0
Can_Ab_Bro_AEC Pool 4	93.1 ± 0.5 ^d^	89.4 ± 0.2 ^e^	27.7 ± 3.8 ^g^	0
Can_Ab_Bro_AEC Pool 5	45.3 ± 2.1 ^n^	17.9 ± 3.8 ^n^	0	0
Can_Ab_Pap + Bro	82.4 ± 0.6 ^f^	24 ± 2.2 ^l^	10.3 ± 6.2 ^m^	0
Can_Ab_Pap+Bro_Unbound material	61.27 ± 4.8 ^i^	13.21 ± 3.9 °	0	0
Can_Ab_Pap+Bro_AEC Pool 2	34.3 ± 1.9 °	25.8 ± 2.4 ^kl^	0	0
Can_Ab_Pap+Bro_AEC Pool 3	93.6 ± 0.3 ^d^	90.9 ± 0.5 ^d^	19.4 ± 2 ^j^	0
Can_Ab_Pap+Bro_AEC Pool 4	96.4 ± 0.3b ^c^	94.9 ± 0.3 ^b^	56.9 ± 2.1 ^d^	14.3 ± 4.2 ^c^
Can_Ab_Pap+Bro_AEC Pool 5	58.45 ± 2.8 ^j^	21.29 ± 1.2 ^m^	9.8 ± 2.7 ^mn^	0
Liquid_Ab_Pap	93.4 ± 1.1 ^d^	65.2 ± 2.1 ^g^	21.4 ± 0.9 ^i^	5.4 ± 1.1 ^e^
Liquid_Ab_Pap_Unbound material	12.43 ± 1.8 ^r^	0	0	0
Liquid_Ab_Pap_AEC Pool 2	28.3 ± 0.9 ^q^	11.2 ± 0.7 ^p^	0	0
Liquid_Ab_Pap_AEC Pool 3	94.9 ± 0.1 ^cd^	88.5 ± 1.8 ^e^	24.8 ± 2.3 ^h^	0
Liquid_Ab_Pap_AEC Pool 4	98.5 ± 0.1 ^a^	96.3 ± 0.2 ^a^	69.1 ± 2.2 ^c^	13 ± 3.5 ^c^
Liquid_Ab_Pap_AEC Pool 5	57.12 ± 0.7 ^jk^	21.23 ± 0.1 ^m^	10.2 ± 1.1 ^m^	0
Liquid_Ab_ Bro	64.32 ± 1.9 ^h^	21.4 ± 0.4 ^m^	10.2 ± 1.9 ^mn^	0
Liquid_Ab_Bro_Unbound material	14.26 ± 4.9 ^r^	2.7 ± 1.9 ^r^	0	0
Liquid_Ab_Bro_AEC Pool 3	34.68 ± 0.8 °	11.2 ± 2.9 ^p^	0	0
Liquid_Ab_Bro_AEC Pool 4	52.9 ± 0.7 ^l^	34.7 ± 1.9 ^i^	10.21 ± 0.8 ^mn^	0
Liquid_Ab_Bro_AEC Pool 5	64.98 ± 1.8 ^h^	38.9 ± 0.7 ^h^	9.7 ± 1.9 ^n^	1.1 ± 1.8 ^f^
Liquid_Ab_Pap+Bro	93.1 ± 0.8 ^d^	26 ± 3.5 ^jk^	11.4 ± 10.2 ^l^	0
Liquid_Ab_Pap+Bro_AEC Pool 2	32.1 ± 0.6 ^p^	20.6 ± 3.5 ^m^	3.1 ± 4.1 °	0
Liquid_Ab_Pap+Bro_AEC Pool 3	95.3 ± 0.3 ^cd^	93.6 ± 1 ^c^	47.3 ± 0.8 ^e^	2.8 ± 2.2 ^ef^
Liquid_Ab_Pap+Bro_AEC Pool 4	98.4 ± 0.1 ^a^	96.1 ± 2.2 ^a^	92.4 ± 1.2 ^a^	25.7 ± 0.4 ^a^
Liquid_Ab_Pap+Bro_AEC Pool 5	47.4 ± 2.9 ^m^	21.49 ± 4.1 ^m^	1.4 ± 2.9 ^q^	0
Heparin Standard	16	4	2	0.5
	91.5 ± 0.6 ^a^	75.0 ± 1.3 ^b^	48.0 ± 2.1 ^c^	27.6 ± 1.2 ^d^

“Ab” stands for abalone, “Pap” stands for papain enzyme, “Bro” stands for bromelain enzyme while “Pap+Bro” reflects the combination of both papain and bromelain enzymes. Alphabetic letters shows the difference between different samples and treatments, ANOVA *p* < 0.05.

**Table 4:** Abalone extracts and AEC pools and thromboelastography.

Sample Description	SP Conc. (μg/mL)	R (s)	MA (mm)	α (Degree)
Control Saline	0	445 ± 14.5	55.2 ± 1.2	45.2 ± 0.5
Can_Ab_Pap	20	760 ± 20.5 **	37.6 ± 2.4 **	12 ± 1.5 **
	80	1115 ± 21.8 **	33.3 ± 1.7 **	22.2 ± 5.4 **
Can_Ab_Pap_AEC Pool 3	20	770 ± 8.5 **	35.3 ± 2.3 **	12 ± 1.7 **
	30	1245 ± 12.2 **	34.8 ± 0.5 **	15.4 ± 0.9 **
Can_Ab_Pap_AEC Pool 4	22	930 ± 10.6 **	34.4 ± 1.9 **	12.8 ± 0.8 **
	34	1475 ± 25.5 **	24.9 ± 1.5 **	10 ± 0.9 **
Can_Ab_Bro	10	635 ± 10.2 **	49.1 ± 2.5 **	23.1 ± 3.7 **
	60	1010 ± 24.7 **	29.8 ± 1.9 **	5.6 ± 4.1 **
Can_Ab_Bro_AEC Pool 3	7	515 ± 15.6 **	40.2 ± 3.6 **	31.7 ± 2.1 **
	35	915 ± 20.3 **	33.4 ± 2.8 **	12.5 ± 1.7 **
Can_Ab_Bro_AEC Pool 4	8	495 ± 4.9 **	27 ± 0.5 **	18.1 ± 0.6 **
	38	645 ± 15.6 **	33.6 ± 1.2 **	26 ± 0.9 **
Can_Ab_Pap+Bro	20	845 ± 12.8 **	41.6 ± 1.4 **	19 ± 2.1 **
	70	1340 ± 24.7 **	37.3 ± 2.5 **	14.3 ± 1.9 **
Can_Ab_Pap+Bro_AEC Pool 3	15	810 ± 20.3 **	37.3 ± 4.9 **	17.4 ± 1.1 **
	31	1180 ± 25.9 **	35.6 ± 1.6 **	16.6 ± 0.4 **
Can_Ab_Pap+Bro_AEC Pool 4	13	690 ± 6.5 **	28.5 ± 1.4 **	17.2 ± 0.2 **
	25	855 ± 12.5 **	21.7 ± 1.8 **	20.8 ± 0.4 **
Liquid_Ab_Pap	30	940 ± 16.5 **	34.5 ± 4.4 **	12.5 ± 4.7 **
	50	1410 ± 35.2 **	31.2 ± 1.2 **	14.9 ± 1.2 **
Liquid_Ab_Pap_AEC Pool 3	8	670 ± 14.7 **	46.3 ± 6.5 **	26.9 ± 4.9 **
	39	1540 ± 23.6 **	33.6 ± 3.9 **	13.6 ± 3.4 **
Liquid_Ab_Pap_AEC Pool 4	10	500 ± 6.9 **	33.8 ± 2.1 **	28.8 ± 0.2 **
	51	705 ± 11.8 **	25.1 ± 0.5 **	10.2 ± 0.9 **
Liquid_Ab_Bro	40	710 ± 7.5 **	38.9 ± 4.7 **	25.9 ± 2.8 **
	90	1075 ± 12.5 **	31.5 ± 1.2 **	12.2 ± 3.1 **
Liquid_Ab_Bro_AEC Pool 4	32	990 ± 4.2 **	36.1 ± 1.9 **	35.7 ± 1.7 **
	64	1295 ± 10.9 **	26.9 ± 2.9 **	17.5 ± 2.1 **
Liquid_Ab_Bro_AEC Pool 5	4	620 ± 27.1 **	36.7 ± 5.9 **	32.3 ± 3.5 **
	19	1115 ± 12.8 **	33.8 ± 7.6 **	13.7 ± 4.7 **
Liquid_Ab_Pap+Bro	20	890 ± 8.5 **	39.6 ± 3.2 **	23.5 ± 1.9 **
	40	1495 ± 24.5 **	36.1 ± 1.9 **	12.4 ± 2.2 **
Liquid_Ab_Pap+Bro_AEC Pool 3	13	685 ± 14.3 **	43 ± 2.9 **	29.6 ± 4.1 **
	26	1865 ± 20.5 **	31.8 ± 3.9 **	8.5 ± 3.2 **
Liquid_Ab_Pap+Bro_AEC Pool 4	9	650 ± 10.3 **	27.3 ± 1.2 **	27.7 ± 0.8 **
	45	1420 ± 15.8 **	35.6 ± 0.9 **	15.2 ± 0.1 **

“SP” stands for Sulphated Polysaccharides “Ab” stands for abalone, “Pap” stands for papain enzyme, “Bro” stands for bromelain enzyme while “Pap+Bro” reflects the combination of both papain and bromelain enzymes. ** Using the Dunnett’s Multiple Comparison Test, all the treatment are significantly different to each other by comparing with saline control.
